# microRNA Expression Profiles in the Ventral Hippocampus during Pubertal Development and the Impact of Peri-Pubertal Binge Alcohol Exposure

**DOI:** 10.3390/ncrna5010021

**Published:** 2019-03-05

**Authors:** AnnaDorothea Asimes, Chun K. Kim, Yathindar S. Rao, Kyle Bartelt, Toni R. Pak

**Affiliations:** Loyola University Chicago Stritch School of Medicine, Department of Cell and Molecular Physiology, Maywood, IL 60153, USA; aasimes@luc.edu (A.A.); ckim9@luc.edu (C.K.K.); yathirao@gmail.com (Y.S.R.); kbartelt@luc.edu (K.B.)

**Keywords:** adolescence, microRNA, neurodevelopment, gene regulation, alcohol, puberty

## Abstract

Adolescence is hallmarked by two parallel processes of sexual maturation and adult patterning of the brain. Therefore, adolescence represents a vulnerable postnatal period for neurodevelopment where exogenous factors can negatively impact adult brain function. For example, alcohol exposure during pubertal development can lead to long-term and widespread neurobiological dysfunction and these effects have been shown to persist even in the absence of future alcohol exposure. However, the molecular mechanisms mediating the persistent effects of alcohol are unclear. We propose that dysregulation of microRNAs (miR) could be a unifying epigenetic mechanism underlying these widespread long-term changes. We tested the hypothesis that repeated alcohol exposure during pubertal development would cause disruption of normal miR expression profiles during puberty and, subsequently, their downstream mRNA target genes in the ventral hippocampus using an established rat model of adolescent binge drinking. We found 6 alcohol-sensitive miRs that were all downregulated following alcohol exposure and we also investigated the normal age-dependent changes in those miRs throughout the pubertal period. Interestingly, these miRs were normally decreased throughout the process of puberty, but alcohol prematurely exacerbated the normal decline in miR expression levels. The work presented herein provides foundational knowledge about the expression patterns of miRs during this critical period of neurodevelopment. Further, this regulation of miR and mRNA expression by alcohol exposure presents a complex regulatory mechanism by which perturbation in this time-sensitive period could lead to long-term neurological consequences.

## 1. Introduction

Adolescence is an important period of human brain development, where the neural circuitry governing executive function and adult-like responses to stressors is still being shaped. The timing of adolescence coincides with sexual maturation, yet brain development extends beyond the period when sexual maturation is complete. These two major developmental processes are of critical importance to long-term cognitive health and involve complicated, and yet understudied, programs of gene regulation. Adolescence is a particularly vulnerable developmental period, as drug use or exposure to physical and/or psychological stressors can disrupt both neurological development and sexual maturation. Unfortunately, teenage culture often involves increased participation in risky behaviors, such as binge alcohol drinking. Over 90% of all alcohol consumed by underage Americans is done so in a binge pattern, which is defined by the National Institute on Alcohol Abuse and Alcoholism (NIAAA) as raising the blood alcohol concentration (BAC) above 0.08% within 2 h. Binge alcohol consumption among adolescents is a major health concern, with 21% of teenagers in the United States reporting binge-pattern drinking behavior in the last 30 days. This behavior is not only dangerous at the time of exposure but can also lead to various health problems in adulthood such as increased risk for developing depression, mood disorders, alcohol dependence, and neurodegenerative diseases [1,2,3,4].

Non-human research models have shown that pubertal alcohol exposure has detrimental effects on the brain that persist into adulthood. For instance, there is a decrease in myelination of the prefrontal cortex in animals exposed to alcohol during pubertal development, and the amount of myelin does not ever reach normal adult levels as the animals age [5]. Others have shown that there is a lower threshold of activation for neurons in the CA1 region of the hippocampus following adolescent exposure to alcohol, and this leads to decreased complexity in dendritic arborization [6]. These symptoms of adolescent alcohol exposure last long into adulthood even in the absence of continued alcohol use, suggesting a stable change in neuronal function, most likely due to improper synaptic patterning. Our lab has established a rat model of repeated binge alcohol exposure during puberty that closely mimics both the timing of intoxication (one bout per day via oral gavage of 3 g/kg alcohol) and blood alcohol levels reached by teens who engage in binge drinking [7,8,9,10,11]. Importantly our model does not include training or addiction to alcohol consumption nor chronic exposure, therefore there are no prolonged/severe withdrawal symptoms, indicating that the animals did not become alcohol dependent. These characteristics, therefore, make this a well-fit rodent model to investigate the underlying molecular mechanisms that contribute to the negative effects of teenage binge-pattern alcohol consumption on brain and sexual development. We have used this model to study the long-term effects of adolescent binge alcohol consumption in the hypothalamus and found persistent changes in the adult male stress response, even in the absence of further alcohol exposure [7,9,12]. We have also examined the impact of this paradigm on future offspring, and found both maternal and paternal alcohol exposure (preconception) altered offspring DNA methylation patterns and development [10,11]. These widespread and long-lasting changes, on both a molecular and behavioral level, suggest the possibility of a common underlying molecular mechanism. These changes also appear to be sex-specific, with a larger vulnerability to alcohol in males, specifically altering genes that regulate the physiological stress response. Therefore, the current study focused on gene expression changes in the ventral hippocampus of males, which plays a central role in the maturation of the stress response that occurs during puberty. We hypothesized that alcohol-induced changes in non-coding RNAs could provide an epigenetic mechanism that would perpetuate the effects of pubertal binge alcohol exposure into adulthood. 

MicroRNAs (miR)s are small non-coding RNAs that regulate gene expression at the post-transcriptional level by inhibiting translation of target mRNAs. A single miR can regulate many mRNA targets and each mRNA can be targeted by multiple miRs, thereby creating complex regulatory networks that are responsive to acute extra- and intracellular signals. It is well known that miR biogenesis and mature miR expression are under precise control and are central to neurodevelopment. Deletion of key miR biogenesis enzymes, such as drosha and dicer, cause global disruptions in miR function resulting in brain deformation and death [13,14]. On the other hand, specific miRs, such as miR-124 and miR-9, are important regulators of neuronal development [15,16,17,18,19,20,21,22] and, along with other miRs, have critical roles in regulating synaptic plasticity [23,24,25,26,27,28,29,30,31,32]. 

Previous work in our lab demonstrated that the expression of several miRs was altered in our repeated binge alcohol exposure animal paradigm, both immediately following alcohol exposure and after a period of prolonged abstinence [8]. Moreover, we showed that normal pubertal development (i.e., in the absence of alcohol) was also accompanied by changes in the expression pattern of these specific miRs, leaving this developmental program potentially vulnerable to alteration by alcohol. With advancements in our knowledge of the complexity of miR regulatory networks, we took a larger scale approach to investigate the effect of repeated ethanol exposure on miRs that are known to be central to neurodevelopment and the downstream impact on their regulation of target genes. We hypothesized that repeated adolescent binge alcohol exposure would alter the expression of genes related to neurodevelopment by dysregulating the normal temporal pattern of miR expression in the ventral hippocampus. We investigated these questions using our well-established animal treatment paradigm of repeated binge alcohol exposure during pubertal development in male Wistar rats. Through careful examination of the normal developmental profiles in this animal model, along with use of both wide-ranging and focused gene expression analyses, we determined the normal expression patterns of alcohol-sensitive miRs through puberty and investigated the effect of alcohol exposure on this developmental program. 

## 2. Results

### 2.1. Experiment 1: Hormone Levels and Spermatogenesis in Untreated Male Wistar Rats at Post-Natal Day (PND) 30, 44, and 74

**Figure 1:**
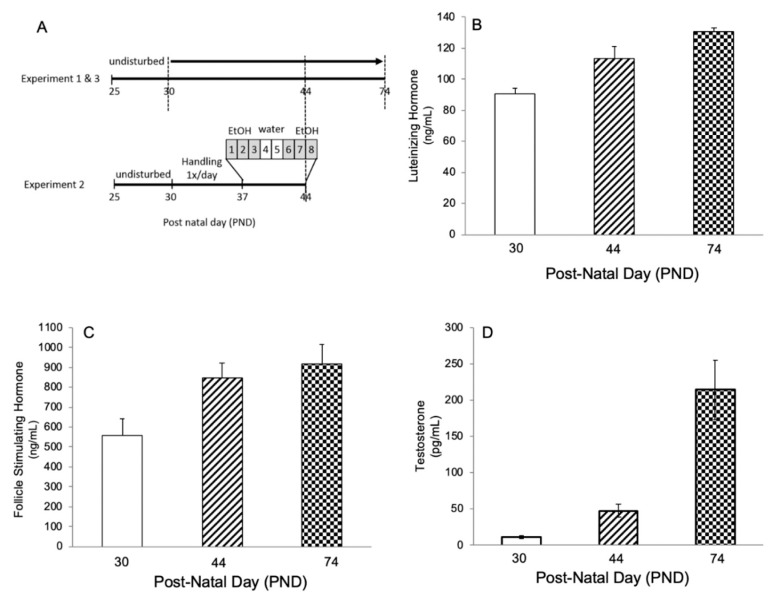
Animal paradigm and hormone levels across pubertal development. (**A**) Animal paradigm for Experiment 1 (untreated) and Experiment 2 (repeated binge ethanol exposure). Dotted lines indicate day of euthanasia. Plasma hormone levels of (**B**) luteinizing hormone (LH), (**C**) follicle-stimulating hormone (FSH), and (**D**) testosterone (T) in pre-, peri- and post-pubertal male rats (post-natal day (PND) 30, 44, and 74, respectively). Data are depicted as mean ± SEM, N = 10/age group.

In order to confirm the concurrent timing of neuronal development and pubertal maturation, we first measured hormone and sperm maturation profiles throughout our paradigm window. Our well-established paradigm of repeated binge alcohol exposure extends from PND 30 (handling begins), with a midpoint of PND 44 (final alcohol dose) through PND 74 (post-pubertal euthanizing time point), therefore we chose these dates to investigate here (Figure 1A). Plasma levels of luteinizing hormone (LH), follicle stimulating hormone (FSH), and testosterone (T) were measured in untreated male Wistar rats at PND 30, 44, and 74 to ensure that our binge alcohol paradigm accurately targets a time period that corresponds with the physiological hallmarks of sexual maturation. LH, FSH, and T levels in the plasma all increased progressively from PND 30, 44, to 74 (Figure 1B–D). Moreover, the values were within the normal physiological ranges for juvenile and adult male rats [33]. 

**Figure 2:**
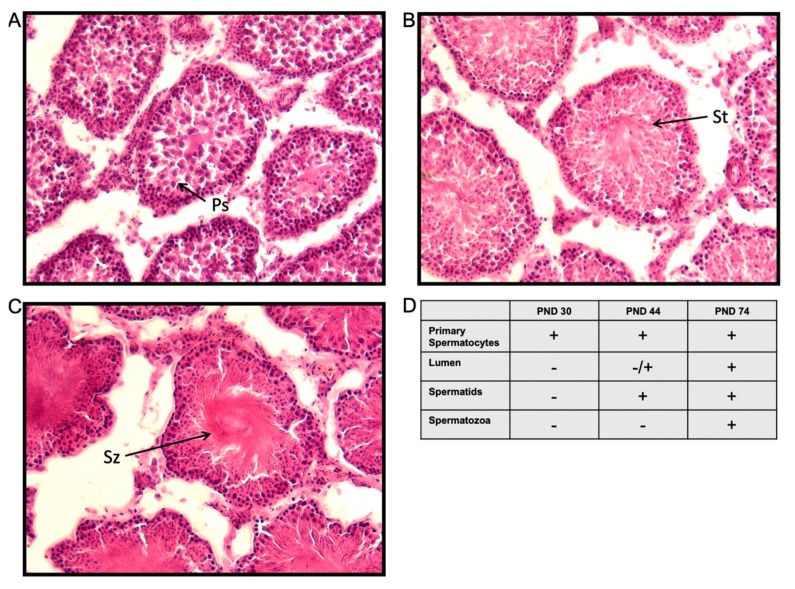
Representative images of testes morphology across male pubertal development. Histological cross-sections of testes stained with hematoxylin and eosin at (**A**) PND 30, (**B**) PND 44, and (**C**) PND 74 (N = 3/ age group). Arrows point to primary (Ps) spermatocytes, spermatids (St) and spermatozoa (Sz). (**D**) Table summarizing morphological differences in testes between PND 30, PND 44, and PND 74. Morphological markers are indicated as present (+) or absent (−) in 100% of animals within an age group. Morphological markers present in less than 100% of animals within an age group indicated as (−/+).

We also measured morphological markers of spermatogenesis in the testes of these males to assess the progression of sexual maturation. Spermatogonia and primary spermatocytes were visible in testes taken from PND 30 animals; however, as expected, a defined lumen and spermatids were absent in all animals at that age (Figure 2D). Differentiation of round and elongated spermatids was evident in PND 44 testes, and a defined lumen was visible in some, but not all, seminiferous tubule cross sections (Figure 2B,D). These data represent the degree of heterogeneity among the animals in the progression of sexual maturation at the peri-pubertal time point (PND 44). A clearly defined lumen was present in all seminiferous tubule cross sections and there was a noticeable abundance of apparent mature spermatozoa in all animals by PND 74 (Figure 2C).

### 2.2. Experiment 2: Repeated Binge Alcohol during Pubertal Development Alters the Neurodevelopmental Gene Network in the Ventral Hippocampus

**Figure 3:**
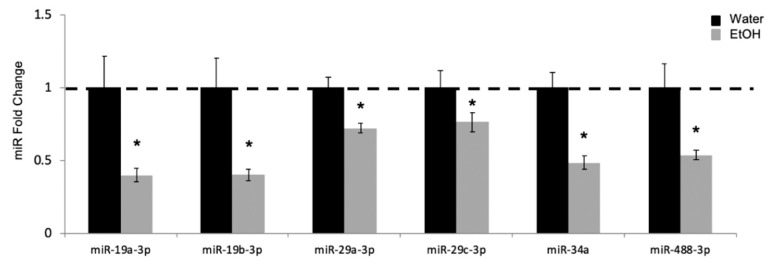
Ethanol treatment during peri-puberty alters microRNA (miR) expression at PND 44. Effects of repeated binge-pattern EtOH exposure on miR levels in the ventral hippocampus of male rats at PND 44 following repeated binge-pattern EtOH administration paradigm during peri-puberty. Data analyzed by ^ΔΔ^Ct method and depicted as mean ± SEM. N = 10/group, an (*) indicates *p* < 0.05.

Having confirmed the timing of maturation in our animal model, we next wanted to assess the impact of peri-pubertal alcohol exposure on miR expression during this period of neurodevelopment. Briefly, our repeated binge alcohol paradigm used discrete and moderate doses of alcohol throughout puberty [7,8,9]. Starting at PND 37, male Wistar rats were given 3 g alcohol/kg body weight (20% *v*/*v* solution) via oral gavage once per day for 3 days, followed by 2 days tap water and another 3 days alcohol (Figure 1A). Control groups received equal volumes of tap water for all 8 days, and all animals were euthanized one hour after the last gavage on PND 44. Our previous work using this paradigm showed long-term alterations to the expression of specific miRs targeting BDNF (brain-derived neurotrophic factor), a critical regulator of many neurodevelopmental processes, in the ventral hippocampus. Therefore, in this study we performed a qPCR-based platform array (N = 3/group) to broadly screen for miRs that are known to target other genes important for neurodevelopment (full list of miRs in Table S1). Our results demonstrated that only 6 miRs (miR-19a-3p, miR-19b-3p, miR-29a-3p, miR-29c-3p, miR-34a, and miR-488-3p), out of a total 88 measured on the array, were significantly altered in animals exposed to repeated binge alcohol compared to control-treated counterparts. These 6 miRs were then further validated using the full cohort of animals with RT-qPCR (N = 10/ group, Figure 3). Notably, levels of miR-19a/b-3p, miR-34a-3p, and miR-488-3p were decreased more than two-fold, whereas miR-29a/c-3p levels were decreased ~30% compared to the control animals (Figure 3).

**Figure 4:**
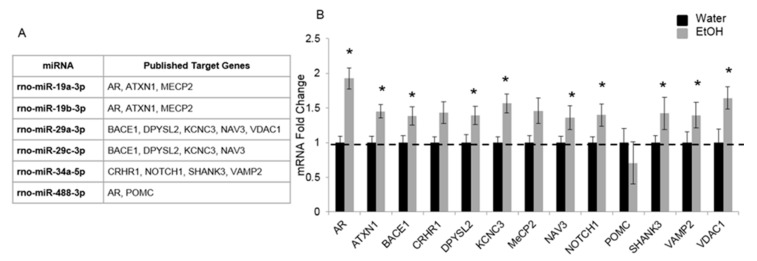

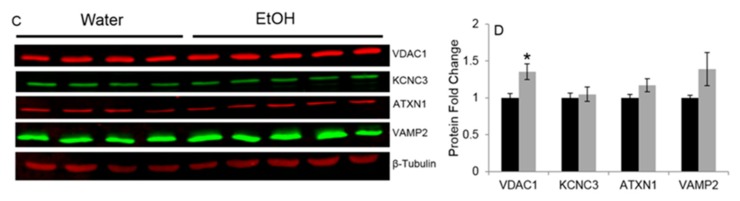
Ethanol treatment during peri-puberty alters validated mRNA targets of EtOH-sensitive microRNAs (miRs) at PND 44. (**A**) Table of published validated mRNA targets for identified EtOH-sensitive miRs. (**B**) mRNA expression levels in the ventral hippocampus of male rats at PND 44 following repeated binge-pattern EtOH administration paradigm during peri-puberty. Data analyzed by ^ΔΔ^Ct method and depicted as mean ± SEM. N = 10/group, an (*) indicates *p* < 0.05. (**C**) Western blot and (**D**) relative quantification of protein expression in ventral hippocampus tissue of the target mRNAs increased by EtOH. Data depicted as relative mean compared to β-Tubulin ± SEM, N = 5/group, an (*) indicates *p* < 0.05.

To assess the potential functional role of these alcohol-sensitive miRs, we generated a list of genes from the literature that were validated targets of each miR that our results demonstrated were significantly altered by pubertal alcohol exposure (Figure 4A). This list of target genes was further refined based on their previously characterized roles in brain development, synaptic plasticity and/or pubertal maturation. Each mRNA target gene from this list was then quantified in the ventral hippocampus by RT-qPCR in our animal model. Overall, we tested 13 target mRNAs using RT-qPCR and found that 12 of them were significantly increased in the ventral hippocampus of animals exposed to repeated binge alcohol compared to water treated controls (Figure 4B). In general, the mRNA levels for each gene measured were increased, which would be predicted given our observed decreases in their putative miR regulators (Figure 3). Notably, androgen receptor (AR) mRNA was increased by 2-fold, and, interestingly, it is a verified target of 3 of the 6 miRs affected by binge alcohol. There was an upward trend in CRHR1 and MeCP2 mRNA expression, yet due to the considerable variability often observed in this outbred strain of rats, these increases were not statistically different from water controls (Figure 4B). Due to limited remaining tissue samples, 4 genes with the highest mRNA enrichment in our alcohol-treated animals were selected for protein measurements: ATXN1 (1.45-fold), KCNC3 (1.57-fold), VAMP2 (1.39-fold), and VDAC1 (1.64-fold). Despite significant changes in mRNA expression, VDAC1 was the only alcohol-miR target that showed statistically significant increase (*p* < 0.05) at the protein level following repeated binge alcohol exposure (1.35 fold-increase, normalized to β-tubulin, Figure 4C,D). 

**Figure 5:**
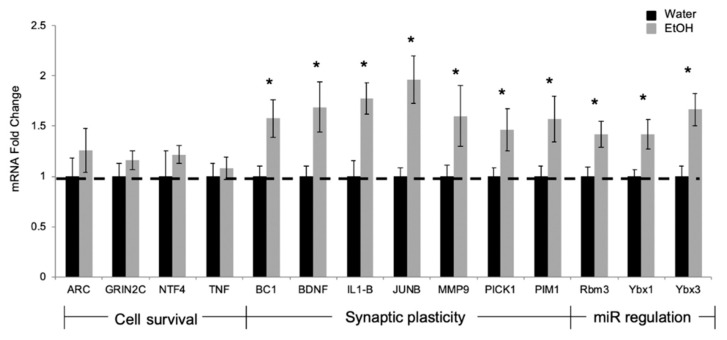
Ethanol treatment during peri-puberty alters mRNA expression at PND 44. Effects of repeated binge-pattern EtOH exposure on mRNA levels in the ventral hippocampus of male rats at PND 44 following repeated binge-pattern EtOH administration paradigm during peri-puberty. Genes grouped by function, listed below the X-axis. Data analyzed by ^ΔΔ^Ct method and depicted as mean ± SEM. N = 10/group, an (*) indicates *p* < 0.05.

Outside of neonatal development, puberty is one of the most critical developmental stages for synaptic strengthening and pruning. Therefore, our next experiment investigated the effects of repeated binge alcohol exposure on mRNA levels for genes known to be associated with synaptic plasticity, but that were not verified targets of the miRs we identified as alcohol-responsive. We used a qPCR-based platform array (N = 3/group) containing probes for 84 genes known to be crucial for neurodevelopmental processes (full list shown in Table S2). These included several early response genes, genes that mediate synaptic plasticity and memory formation, as well as genes that are required for neurotransmission and neuronal connectivity. This initial screen identified 14 genes that were alcohol-responsive, which we then validated by RT-qPCR using the full cohort of animals (N = 10/group, Figure 5). 

The majority of significantly different genes observed on this broad qPCR platform screen were consistent upon validation with the exception of 4 genes, many of which are involved in cell survival: activity-regulated cytoskeletal associated protein (*Arc*), glutamate ionotropic receptor NMDA type subunit C (*Grin2c*), neurotrophin 4 (*Ntf4*), and tumor necrosis factor (*Tnf*) (Figure 5). On the other hand, 10 genes were significantly increased following adolescent binge alcohol exposure and 6 of those are known regulators of synaptic plasticity including brain cytoplasmic RNA 1 (*Bc1*), brain-derived neurotrophic factor (*Bdnf*), interleukin 1-β (*IL-1β*), AP-1 transcription factor subunit B (*JunB*), protein interacting with C kinase 1 (*Pick1*), serine/threonine-protein kinase Pim-1 (*Pim1*), and matrix metallopeptidase 9 (*Mmp9*) (Figure 5). Interestingly, 3 of the genes that were significantly increased by binge alcohol exposure are nucleic acid binding proteins with proposed roles in the regulation of miR biogenesis including RNA binding motif protein 3 (*Rbm3*), Y-box binding protein 1 (*Ybx1*), and Y-box binding protein 3 (*Ybx3*) [34,35]. Taken together, these data indicate that pubertal alcohol exposure can alter the regulation of mRNAs and miRs, which could impact neurodevelopment and synaptic plasticity during puberty.

### 2.3. Experiment 3: Temporal Regulation of Alcohol-Sensitive miRs Through Puberty 

**Figure 6:**
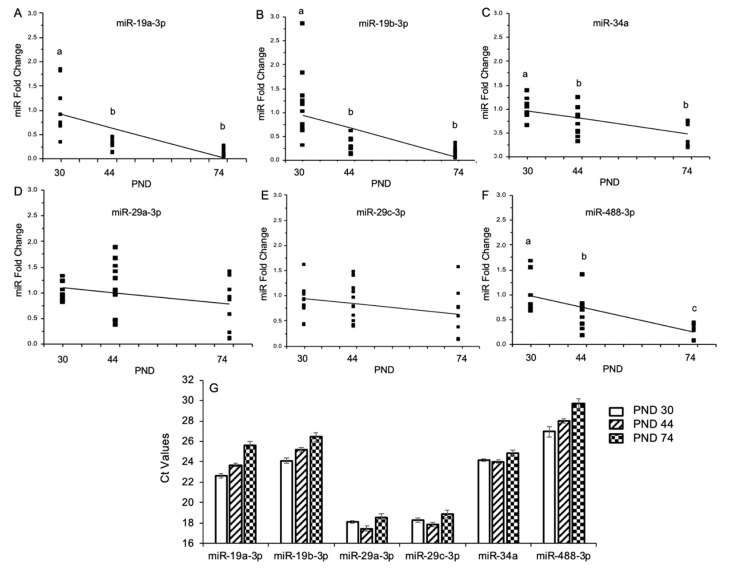
microRNA (miR) expression profile in the ventral hippocampus during male pubertal development. (**A**–**F**) Fold change differences in mature miR expression levels at PND 30, 44, and 74 as analyzed by ^ΔΔ^Ct method. Data are depicted as a scatter plot for each animal in each group, overlaid with a line of best fit. Dissimilar lower-case letters indicate statistically significant difference between groups. (**G**) Raw qPCR Ct values of mature miRs demonstrating relative abundance in the ventral hippocampus across pubertal development. Data are depicted as mean ± SEM. N = 10/age group Ct = cycle threshold.

Next, we quantified the normal (non-manipulated) temporal pattern of expression for the 6 alcohol-sensitive miRs identified in Experiment 2. miR expression was measured in the ventral hippocampus of animals from Experiment 1 (see Figure 1A) using RT-qPCR in untreated animals at pre-puberty (PND 30), peri-puberty (PND 44), and early adulthood (PND 74) (N = 10/age group). The majority of miRs that we measured showed a step-wise decrease in expression over the time course of sexual maturation (Figure 6A–F). A notable exception was seen with the closely related mature miR species, miR 29a-3p, and 29c-3p, which showed no significant reduction in expression during pubertal progression (Figure 6D,E). A comparison of the raw cycle threshold (Ct) values allowed us to determine the abundance of expression of these miRs during this time period. In addition to having a unique developmental profile, miR-29a-3p and miR-29c-3p were the most highly expressed miRs in the ventral hippocampus at all time points tested, whereas miR-488-3p was the lowest expressed miR (Figure 6G). 

## 3. Discussion

Together, these experiments provide novel foundational data that describe miR expression changes in the ventral hippocampus across different stages of pubertal development. In addition, the data show a strong correlation between alcohol-induced changes in these miRs at peri-puberty and the expression levels of their mRNA targets, many of which are directly implicated in the regulation of sexual maturation and/or neuronal development. miRs have the ability to regulate hundreds of mRNA gene targets, which in turn can be regulated by multiple miRs; therefore, this work provides fundamental groundwork towards uncovering potentially broad cellular regulatory programs that can be disrupted by the extremely popular teenage behavior of binge drinking. 

There have been only a handful of studies to date investigating the role of miRs in the brain during pubertal development. To our knowledge, this study and our previous publication [8] were the first to measure normal changes in miR expression in the brain at several time points and in multiple brain regions during the pubertal transition, as other studies have been limited to measuring miR expression at either the juvenile (pre) or adult (post) endpoints. These normal developmental expression profiles form a critical baseline that allows us to better understand the function of these miRs during adolescent brain development and the potential consequences of repeated binge alcohol exposure during this developmental transition. Our data are in agreement with other studies demonstrating that miR expression in the brain changes with age [8,36]. Specifically, the expression of miR-19a/b-3p, miR-34a, and miR-488-3p all progressively decreased from PND 30 to PND 74 in the ventral hippocampus of untreated animals, suggesting that their target genes would be increased and are therefore, likely important mediators of neurodevelopmental processes. The normal decline in miR expression was significantly exacerbated by repeated binge-alcohol administration during peri-puberty indicating that adolescent alcohol exposure prematurely downregulates the expression of miRs in the ventral hippocampus, essentially accelerating their normal temporal regulation through puberty. The progressive increase in plasma hormone concentrations that was observed in our PND 30, 44, and 74 animals were consistent with previous reports in the literature investigating sexual development in male Wistar rats [37]. Recent evidence from other *Rattus norvegicus* strains (Sprague Dawley) approximated the peri-pubertal period to start on PND 32 and end on PND 55, closely corresponding to our treatment window [38]. Taken together, these data confirm that the binge alcohol treatment paradigm used accurately targets the time period coinciding with pubertal development in male Wistar rats, and that adolescent alcohol exposure impacts normal developmental patterns of miR expression. 

Previously, we demonstrated that adolescent binge alcohol exposure altered the expression of several miRs in a brain-region specific manner at PND 44 [8]. Notably, these miRs remained dysregulated for at least one month after the last exposure to alcohol. Blood alcohol levels were undetectable by 6 h after the last dose in our repeated binge-pattern paradigm (unpublished data), suggesting that these long-term changes in miR expression could be due to permanent modifications of biogenesis pathways. Indeed, we showed that alcohol altered both Drosha and Dicer, two RNase III enzymes that are required for the processing of primary-to-mature miRs [8]. However, changes in these critical miR biogenesis enzymes would be expected to have a global impact on all miRs in the cell, which is in contrast to the select few that we have observed being altered by alcohol exposure. Therefore, it is likely that alcohol induces specific modifications to select miR transcripts, or that other cellular mechanisms are able to protect some, but not all, miRs. Future studies in our laboratory are ongoing to determine the molecular mechanisms by which alcohol induces specific long-term changes in miR expression in the brain.

The ventral hippocampus is a brain region that is broadly characterized for mediating learning and memory formation, emotional memory, and mood. Further, neuronal projections from the hippocampus are intimately connected to the hypothalamus to coincidently regulate a variety of other physiological processes, including sexual maturation. The alcohol-regulated miRs we have identified in this study (miR-19a/b-3p, miR-29a/c-3p, and miR-34a) have been shown to play important roles in neurogenesis and neuronal development [39,40,41,42,43,44]. Specifically, miR-19a-3p and miR19b-3p are important modulators of neural progenitor cell migration [45], whereas overexpression of miR-34a promoted cell proliferation and improved learning in vivo [39]. Notably, miR-19a has also been reported to post-transcriptionally inhibit *Atxn1*, which codes for a DNA-binding protein that becomes neurotoxic upon accumulation. Inhibition of miR-19a increased cellular levels of *Atxn1* and, consequently, reduced cell viability [46]. Therefore, the premature reduction in miR-19a/b-3p, miR-29a/c-3p, and miR-34 caused by repeated alcohol exposure could result in altered neurogenesis during puberty that would be potentially irreversible. Similar to our study, Lobouesse and colleagues recently demonstrated that consumption of a high fat diet during adolescence significantly altered 38 miRs in the prefrontal cortex, a brain region important for mediating cognitive and executive functions [47]. In that study, miRs regulating genes important for axon guidance were significantly affected, suggesting that neuronal connections between functionally aligned brain regions could be disrupted during adolescent brain development. Taken together, these data underscore the vulnerability of the adolescent brain to perturbations such as alcohol use or poor diet and suggest that disruption of miRs during this time period could underlie subsequent neurological deficits in adulthood.

It is important to consider that miR functional analysis is often performed using overexpression or knockout techniques. While these techniques are informative, only the polar ends of the expression spectra are analyzed, making it difficult to draw conclusions concerning moderate deviations from endogenous, baseline expression levels. Therefore, although we observed a significant decrease in the expression of 6 miRs with previously characterized roles in neurodevelopment and disease, it is unclear whether these changes alone mediate the neurotoxic effects of alcohol. An interesting alternative is that while the downregulation of these miRs itself does not elicit an immediate phenotype, it could prime the cellular environment such that it is more prone to neurodegeneration following a subsequent insult. Alternatively, the possibility remains that the cumulative dysregulation of multiple miRs may synergistically elicit a deleterious phenotype, or a protracted cascade effect that impacts mRNAs that are not direct miR targets. Indeed, our data demonstrated that alcohol altered the expression of several mRNAs that were not predicted targets of the alcohol-sensitive miRs. In addition, mRNA dysregulation was not necessarily correlated with protein levels in our tissue. The direct relationships between miR, mRNA, and protein are not as straightforward or stoichiometric as they may seem, and the interplay between these regulatory elements also occurs on a different timescale than the snapshot we are able to see in our current paradigm. Slight changes at any stage of gene regulation can potentially have long-lasting effects on its function, and further studies into the typical relationship between these elements will allow us to study perturbations to this system more in depth. While behavioral and cellular deficits were not investigated in the current study, the effects of adolescent binge drinking on these endpoints have been thoroughly documented in the literature [5,6,7,48,49]. Moreover, our prior studies showed that adolescent binge drinking followed by exposure to an acute psychological stressor in adulthood (i.e., restraint stress) significantly increased risk assessment behaviors [9]. These data raise the possibility of functional consequences on the maturation of hippocampal-mediated cognitive and emotional processes, such as memory formation and anxiety response, immediately in adolescence and into adulthood. 

Adolescence represents a critical period of brain development where new synaptic connections are formed, and unused connections pruned away [50]. Specifically, in the hippocampus, thousands of neurons are formed and turned over during puberty to create the necessary adult neuronal patterning [51]. Therefore, this period of life is particularly vulnerable to exogenous stressors, such as repeated binge alcohol exposure, which can elicit both immediate deleterious effects on synaptic pruning and long-term neuronal function. We propose in this work that a central mechanism underlying these changes is a disruption in the normal age-related profile of miR expression. This study characterized the normal expression pattern of miRs in the ventral hippocampus that are associated with synaptic plasticity, as well as the impact of repeated binge-alcohol exposure on this developmental program. Overall, we find that alcohol caused premature downregulation of several miRs and that altered expression of these master regulators could have downstream effects on gene programs necessary to form correct synaptic connections. 

## 4. Materials and Methods

### 4.1. Animals

Male Wistar rats were purchased from Charles River Laboratories (Wilmington, MA, USA) at post-natal day (PND) 25 and were allowed to acclimate for 5 days after arrival. Animals were pair housed on a 12:12 light/dark cycle. Food and water were available *ad libitum* throughout the experimental paradigm. All procedures were approved by the Loyola University Chicago Institutional Animal Care and Use Committee (IACUC # 2013034). 

#### 4.1.1. Experiment 1: Normal Pubertal Development (N = 10/age group)

Physiological parameters of sexual maturation (i.e., hormone levels and markers of spermatogenesis) were measured in animals that were not handled or manipulated. Animals arrived at PND 25 and were subjected to normal animal husbandry conditions in the Comparative Animal Facility at Loyola University. Animals were humanely euthanized using inhaled isoflurane followed by rapid decapitation at PND 30, 44, and 74 which correspond to the stages of pre-puberty, peri-puberty, and late-puberty, respectively (Figure 1A). 

#### 4.1.2. Experiment 2: Repeated Binge-Pattern Alcohol Exposure Paradigm (N = 10/treatment)

Animals arrived at PND 25 and were allowed to acclimate under normal housing conditions until PND 30. Animals were then handled 5 min once/day for 7 days to reduce non-specific stress. Animals were held and exposed to the investigator for the first 4 days, after which the oral gavage tool was gradually introduced once/day until PND 36 when animals were given a “dry” oral gavage. Beginning at PND 37, which is defined as peri-puberty in the rat [52], animals were exposed to a repeated binge-pattern alcohol paradigm. This 8-day paradigm has been used previously by our lab and others and is designed to mimic the reported drinking patterns of adolescents [7,53,54]. This pattern of alcohol consumption raises the blood alcohol concentration (BAC) to 150–180 mg/dl in males and 210–240 mg/dl in females without altering body weight or normal growth patterns [7,53]. Animals were given food grade alcohol (Everclear, Luxco, St. Louis, MO, USA) diluted in tap water at a dose of 3 g/kg body weight (20% *v*/*v* solution) or an equal volume of vehicle (water) via oral gavage. Alcohol treatment was given once/day for 3 days, followed by 2 days tap water, and another 3 days alcohol (Figure 1A). Control groups received equal volumes of tap water for all 8 days. All animals were humanely euthanized using inhaled isoflurane followed by rapid decapitation one hour after the last oral gavage on PND 44. 

#### 4.1.3. Experiment 3: Normal Pubertal miR Expression Patterns (N = 10/age group)

Using the same animals as in Experiment 1, we measured mature miR expression levels in the ventral hippocampus at 3 stages of puberty, focusing on the miRs found to be alcohol-sensitive in Experiment 2. Animals were euthanized at PND 30, 44, and 74 which correspond to the stages of pre-puberty, peri-puberty, and late-puberty, respectively (see Figure 1A). 

### 4.2. Tissue Collection

Trunk blood was collected on ice into heparinized tubes at the time of decapitation, centrifuged at 4,500 rpm for 8 min. at 4 °C, and plasma was aliquoted and stored at −20 °C. Testes were immediately removed and flash frozen in isopentane on dry ice, then stored at −80 °C until processed for histological measurements. Brains were immediately removed and freshly dissected in the sagittal plane, separating the right and left hemispheres. The right hemisphere was used to isolate the hippocampus, which was further separated into dorsal and ventral portions (ventral defined as 4.30 to 6.04 mm relative to bregma, according to The Rat Brain in Stereotaxic Coordinates, Fourth Edition Atlas [55]. The ventral hippocampus was placed in Qiazol lysis reagent (Qiagen, Germantown, MD, USA), homogenized via sonication and stored at −80 °C until use. Briefly, brains were sectioned rostral to caudal at 200 μm using a Leica CM3050 S cryostat (Nussloch, Germany) and the ventral hippocampus was microdissected using a Palkovit’s brain punch tool (Stoelting Co., Wooddale, IL, USA). 

### 4.3. Hormone Measurements

Plasma from non-treated animals were used to determine normal circulating levels of luteinizing hormone (LH), follicle stimulating hormone (FSH), and testosterone (T). LH and T ELISA assays were obtained from Enzo Life Sciences (Farmingdale, NY, USA; Catalog # ENZ-KIT107 and ADI-900-065, respectively). The FSH ELISA assay was obtained from LifeSpan Biosciences, Inc (LSBio, Seattle, WA, USA; Catalog # LS-F6305). All assays were performed according to manufacturer instructions. 

### 4.4. Testes Histology and Analysis 

Testes from animals at each pubertal age (N = 3/group) were post-fixed and cryoprotected in sucrose before sectioning on a Leica CM3050 S cryostat at 16 µm thickness. Frozen sections were thaw mounted on Superfrost Plus slides (ThermoFisher Scientific, Waltham, MA, USA) and dried at 37 °C for 30 min, then stained using hematoxylin and eosin (H&E), as previously described [56]. Slides were imaged under a light microscope and images were qualitatively scored by a blinded investigator for the presence of primary spermatocytes, lumen opening, spermatids, and spermatozoa. 

### 4.5. RNA Isolation and Reverse Transcription 

Total RNA was extracted from ventral hippocampus samples using the miRNeasy Mini Kit (Qiagen), according to manufacturer instructions. Genomic DNA was removed using DNA-*free* DNA Removal kit (ThermoFisher Scientific, AM1906). Total RNA (1.0 μg) was used to perform reverse transcription of both miR and mRNA using microScript miR cDNA synthesis kit (Norgen Biotek, #54410, Thorlold, ON, Canada) and High Capacity cDNA Reverse Transcriptase Kit (Applied Biosystems, Foster City, CA, USA), respectively. 

### 4.6. Gene Expression Profiling with Microarray

We used miR and mRNA qPCR-array platforms that had probes targeted to transcripts with known roles in neurodevelopment and neurological disorders in order to understand which miRs and mRNAs are altered after the exposure to repeated binge alcohol. These arrays were miScript miR PCR Array Rat Neurological Development & Disease (cat# MIRN-107Z, Qiagen) and RT Profiler PCR Array Rat Synaptic Plasticity (cat# PARN-126ZA-12, Qiagen) and were carried out according to manufacturer instructions. Target genes of each array are listed in Table S1 (miR) and Table S2 (mRNA). A sample size of 3 per treatment group was used for both arrays. Genes which showed a statistically significant change in expression from the arrays were furthered analyzed with the full sample size (N = 10/group) by RT-qPCR.

### 4.7. Reverse Transcription Quantitative PCR (RT-qPCR)

miR and mRNA RT-qPCR was performed with Fast Start Universal SYBR Green Master Mix (Roche-Genentech, San Francisco, CA, USA) on an Eppendorf Realplex 4 cycler (Hamburg, Germany). Forward primers for specific mature miRs were designed as described in the microScript miR cDNA synthesis handbook and using miRBase 21 as a sequence reference (Table S3). The following program was used for miR RT-qPCR: (1) 95 °C for 10 min, (2) 95 °C for 15 s, (3) 60 °C for 30 s, (4) 72 °C for 15 s, and melting curve analysis (Table S3). The following program was used for mRNA RT-qPCR: (1) 95 °C for 10 min, (2) 95 °C for 15 s, (3) 60 °C for 60 s, and melting curve analysis. Ribosomal protein lateral stalk subunit P1 (Rplp1) and U87 were used to normalize the data for analysis for mRNA and miR RT-qPCR, respectively. miR and mRNA expression was analyzed using the ^ΔΔ^Ct method.

### 4.8. Western Blots

Left hemisphere ventral hippocampus tissue punches were lysed using TPER (ThermoFisher Scientific) and protein lysates were used to determine protein expression of miRNA target genes. Briefly, 30 μg from each sample was run on 10% polyacrylamide gel and then transferred to a PVDF membrane. PVDF blots were then blocked with a 1:1 ratio of 1× PBS and Odyssey Blocking Buffer (Licor, Lincoln, NE, USA) for 1 h. Primary antibodies against proteins of interest (see Table S4) were diluted in 1:1 solution of PBS-T (0.1% tween) and Odyssey Blocking Buffer and incubated overnight at 4 °C then washed and incubated with secondary antibody (see Table S3) in fresh blocking solution (1:1 PBS-T + Odyssey Blocking Buffer) at room temperature for 1 h with shaking. Blots were then rinsed 3× with PBS-T and imaged on Licor Odyssey.

### 4.9. Statistics

Statistical analysis was performed using a one-way ANOVA for endpoints in Experiments 1 and 3, and Fisher’s Least Significant Difference Test for post-hoc analysis where appropriate. Data for Experiment 2 were all compared using two-sample t-test. All analyses were performed using SYSTAT software (Version 13, San Jose, CA, USA) and a statistically significant difference indicated by *p* < 0.05. Error bars = SEM 

## Supplementary Materials

The following are available online at https://www.mdpi.com/2311-553X/5/1/21/s1. Table S1: miR array gene list, Table S2: mRNA array gene list, Table S3: qPCR primers, Table S4: Antibodies.
