# Supplementary material for: microRNA Expression Profiles in the Ventral Hippocampus during Pubertal Development and the Impact of Peri-Pubertal Binge Alcohol Exposure
Source: Noncoding RNA. 2019 Mar 5;5(1):21. doi: 10.3390/ncrna5010021 (PMC6468757; doi:10.3390/ncrna5010021)
Supplement: Supplementary file 1 [file ncrna-05-00021-s001.zip › ncrna-434944-suppl/S1 miRNA array gene list.docx]

| **miRBase or NCBI Accession No.** | **Mature miRNA ID or Gene Symbol** |
| --- | --- |
| MIMAT0000775 | rno-let-7b-5p |
| MIMAT0000776 | rno-let-7c-5p |
| MIMAT0000562 | rno-let-7d-5p |
| MIMAT0000777 | rno-let-7e-5p |
| MIMAT0000779 | rno-let-7i-5p |
| MIMAT0000615 | rno-miR-101b-3p |
| MIMAT0012825 | rno-miR-105 |
| MIMAT0000825 | rno-miR-106b-5p |
| MIMAT0000826 | rno-miR-107-3p |
| MIMAT0000828 | rno-miR-124-3p |
| MIMAT0000830 | rno-miR-125b-5p |
| MIMAT0000831 | rno-miR-126a-5p |
| MIMAT0000834 | rno-miR-128-3p |
| MIMAT0000836 | rno-miR-130a-3p |
| MIMAT0000838 | rno-miR-132-3p |
| MIMAT0003126 | rno-miR-133b-3p |
| MIMAT0000840 | rno-miR-134-5p |
| MIMAT0000844 | rno-miR-138-5p |
| MIMAT0000845 | rno-miR-139-5p |
| MIMAT0000573 | rno-miR-140-5p |
| MIMAT0000849 | rno-miR-143-3p |
| MIMAT0000852 | rno-miR-146a-5p |
| MIMAT0005595 | rno-miR-146b-5p |
| MIMAT0000579 | rno-miR-148b-3p |
| MIMAT0000853 | rno-miR-150-5p |
| MIMAT0000614 | rno-miR-151-3p |
| MIMAT0000854 | rno-miR-152-3p |
| MIMAT0000784 | rno-miR-15b-5p |
| MIMAT0000858 | rno-miR-181a-5p |
| MIMAT0000857 | rno-miR-181c-5p |
| MIMAT0005299 | rno-miR-181d-5p |
| MIMAT0000866 | rno-miR-191a-5p |
| MIMAT0000869 | rno-miR-194-5p |
| MIMAT0000870 | rno-miR-195-5p |
| MIMAT0000789 | rno-miR-19a-3p |
| MIMAT0000788 | rno-miR-19b-3p |
| MIMAT0000876 | rno-miR-203a-3p |
| MIMAT0000602 | rno-miR-20a-5p |
| MIMAT0003211 | rno-miR-20b-5p |
| MIMAT0000883 | rno-miR-212-3p |
| MIMAT0000791 | rno-miR-22-3p |
| MIMAT0000792 | rno-miR-23a-3p |
| MIMAT0000794 | rno-miR-24-3p |
| MIMAT0000797 | rno-miR-26b-5p |
| MIMAT0000799 | rno-miR-27a-3p |
| MIMAT0000800 | rno-miR-28-5p |
| MIMAT0000900 | rno-miR-298-5p |
| MIMAT0000802 | rno-miR-29a-3p |
| MIMAT0000801 | rno-miR-29b-3p |
| MIMAT0000803 | rno-miR-29c-3p |
| MIMAT0000808 | rno-miR-30a-5p |
| MIMAT0000807 | rno-miR-30d-5p |
| MIMAT0000805 | rno-miR-30e-5p |
| MIMAT0000812 | rno-miR-33-5p |
| MIMAT0000577 | rno-miR-337-3p |
| MIMAT0000581 | rno-miR-338-3p |
| MIMAT0000583 | rno-miR-339-5p |
| MIMAT0000589 | rno-miR-342-3p |
| MIMAT0000596 | rno-miR-346 |
| MIMAT0000815 | rno-miR-34a-5p |
| MIMAT0003117 | rno-miR-361-5p |
| MIMAT0003196 | rno-miR-376b-3p |
| MIMAT0003199 | rno-miR-381-3p |
| MIMAT0003201 | rno-miR-382-5p |
| MIMAT0003205 | rno-miR-409a-3p |
| MIMAT0001626 | rno-miR-431 |
| MIMAT0001628 | rno-miR-433-3p |
| MIMAT0005316 | rno-miR-455-5p |
| MIMAT0005319 | rno-miR-484 |
| MIMAT0003203 | rno-miR-485-5p |
| MIMAT0005341 | rno-miR-488-3p |
| MIMAT0003113 | rno-miR-489-3p |
| MIMAT0003381 | rno-miR-499-5p |
| MIMAT0012829 | rno-miR-511-5p |
| MIMAT0003176 | rno-miR-539-5p |
| MIMAT0005325 | rno-miR-598-3p |
| MIMAT0005342 | rno-miR-652-3p |
| MIMAT0000606 | rno-miR-7a-5p |
| MIMAT0000781 | rno-miR-9a-5p |
| MIMAT0004708 | rno-miR-9a-3p |
| MIMAT0000816 | rno-miR-92a-3p |
| MIMAT0005340 | rno-miR-92b-3p |
| MIMAT0000817 | rno-miR-93-5p |
| MIMAT0000819 | rno-miR-98-5p |
| MIMAT0000010 | cel-miR-39-3p |
| MIMAT0000010 | cel-miR-39-3p |
|  | SNORD61 |
|  | SNORD68 |
|  | SNORD72 |
|  | SNORD95 |
|  | SNORD96A |
|  | RNU6-6P |
