# Supplementary material for: microRNA Expression Profiles in the Ventral Hippocampus during Pubertal Development and the Impact of Peri-Pubertal Binge Alcohol Exposure
Source: Noncoding RNA. 2019 Mar 5;5(1):21. doi: 10.3390/ncrna5010021 (PMC6468757; doi:10.3390/ncrna5010021)
Supplement: Supplementary file 1 [file ncrna-05-00021-s001.zip › ncrna-434944-suppl/S2 mRNA array gene list.docx]

| **GeneBank** | **Symbol** | **Description** | **Gene Name** |
| --- | --- | --- | --- |
| NM_019254 | Adam10 | ADAM metallopeptidase domain 10 | MADM |
| NM_001107239 | Adcy1 | Adenylate cyclase 1 (brain) | Ac1 |
| NM_017142 | Adcy8 | Adenylate cyclase 8 (brain) | Ac8 |
| NM_033230 | Akt1 | V-akt murine thymoma viral oncogene homolog 1 | Akt |
| NM_019361 | Arc | Activity-regulated cytoskeleton-associated protein | rg3.1 |
| NM_012513 | Bdnf | Brain-derived neurotrophic factor | - |
| NM_012920 | Camk2a | Calcium/calmodulin-dependent protein kinase II alpha | PK2CDD, PKCCD |
| NM_133605 | Camk2g | Calcium/calmodulin-dependent protein kinase II gamma | - |
| NM_031333 | Cdh2 | Cadherin 2 | N-cadherin |
| NM_024125 | Cebpb | CCAAT/enhancer binding protein (C/EBP), beta | Il6dbp, NF-IL6, TCF5 |
| NM_013154 | Cebpd | CCAAT/enhancer binding protein (C/EBP), delta | C, EBPd, CELF |
| NM_012784 | Cnr1 | Cannabinoid receptor 1 (brain) | SKR6R |
| NM_031017 | Creb1 | CAMP responsive element binding protein 1 | Creb |
| NM_001110860 | Crem | CAMP responsive element modulator | Icer |
| NM_019621 | Dlg4 | Discs, large homolog 4 (Drosophila) | Dlgh4, PSD95, Sap90 |
| NM_012551 | Egr1 | Early growth response 1 | Krox-24, NGFI-A, Ngf1, Ngfi, zif-268 |
| NM_053633 | Egr2 | Early growth response 2 | Krox20 |
| NM_017086 | Egr3 | Early growth response 3 | - |
| NM_019137 | Egr4 | Early growth response 4 | Egr4l1, NGFI-C |
| NM_001127319 | Ephb2 | Eph receptor B2 | RGD1564232 |
| NM_022197 | Fos | FBJ osteosarcoma oncogene | c-fos |
| NM_017295 | Gabra5 | Gamma-aminobutyric acid (GABA) A receptor, alpha 5 | - |
| NM_013145 | Gnai1 | Guanine nucleotide binding protein (G protein), alpha inhibiting 1 | BPGTPB |
| NM_031608 | Gria1 | Glutamate receptor, ionotropic, AMPA 1 | GluA1, gluR-A |
| NM_017261 | Gria2 | Glutamate receptor, ionotropic, AMPA 2 | GluA2, GluR-K2, GluR2, gluR-B |
| NM_032990 | Gria3 | Glutamate receptor, ionotrophic, AMPA 3 | GLUR3, GluA3, GluR-3, GluR-C, GluR-K3 |
| NM_017263 | Gria4 | Glutamate receptor, ionotrophic, AMPA 4 | GluA4, GluR-D, GluR4 |
| NM_017010 | Grin1 | Glutamate receptor, ionotropic, N-methyl D-aspartate 1 | GluN1, NMDAR1, NR1 |
| NM_012573 | Grin2a | Glutamate receptor, ionotropic, N-methyl D-aspartate 2A | GluN2A, NMDAR2A, NR2A |
| NM_012574 | Grin2b | Glutamate receptor, ionotropic, N-methyl D-aspartate 2B | GluN2B |
| NM_012575 | Grin2c | Glutamate receptor, ionotropic, N-methyl D-aspartate 2C | GluN2C, NR2C |
| NM_022797 | Grin2d | Glutamate receptor, ionotropic, N-methyl D-aspartate 2D | GluN2D |
| NM_032069 | Grip1 | Glutamate receptor interacting protein 1 | - |
| NM_017011 | Grm1 | Glutamate receptor, metabotropic 1 | Gprc1a |
| NM_001105711 | Grm2 | Glutamate receptor, metabotropic 2 | - |
| NM_001105712 | Grm3 | Glutamate receptor, metabotropic 3 | mGluR3 |
| NM_022666 | Grm4 | Glutamate receptor, metabotropic 4 | - |
| NM_017012 | Grm5 | Glutamate receptor, metabotropic 5 | mGluR5, mGlur5 |
| NM_031040 | Grm7 | Glutamate receptor, metabotropic 7 | - |
| NM_022202 | Grm8 | Glutamate receptor, metabotropic 8 | Glur8, Gprc1h, Mglur8, mGluR8b, mGlur |
| NM_031707 | Homer1 | Homer homolog 1 (Drosophila) | HOMER1F, Vesl-1 |
| NM_178866 | Igf1 | Insulin-like growth factor 1 | - |
| NM_017128 | Inhba | Inhibin beta-A | - |
| NM_021835 | Jun | Jun oncogene | - |
| NM_021836 | Junb | Jun B proto-oncogene | - |
| NM_031135 | Klf10 | Kruppel-like factor 10 | Tieg |
| NM_053842 | Mapk1 | Mitogen activated protein kinase 1 | ERK-2, ERT1, Erk2, p42-MAPK |
| NM_031055 | Mmp9 | Matrix metallopeptidase 9 | - |
| NM_031521 | Ncam1 | Neural cell adhesion molecule 1 | Cd56, N-CAM, N-CAM-1, NCAM-1, NCAM-C, NCAMC, Ncam |
| NM_001276711 | Nfkb1 | Nuclear factor of kappa light polypeptide gene enhancer in B-cells 1 | EBP-1, NF-kB |
| NM_030867 | Nfkbib | Nuclear factor of kappa light polypeptide gene enhancer in B-cells inhibitor, beta | - |
| NM_001277055 | Ngf | Nerve growth factor (beta polypeptide) | Ngfb, beta-NGF |
| NM_012610 | Ngfr | Nerve growth factor receptor (TNFR superfamily, member 16) | LNGFR, RNNGFRR, Tnfrsf16, p75, p75NTR |
| NM_052799 | Nos1 | Nitric oxide synthase 1, neuronal | bNOS |
| NM_001034199 | Nptx2 | Neuronal pentraxin 2 | NP-II, NP2, Narp |
| NM_024388 | Nr4a1 | Nuclear receptor subfamily 4, group A, member 1 | HMR, Ngfi-b, Nur77 |
| NM_031073 | Ntf3 | Neurotrophin 3 | - |
| NM_013184 | Ntf4 | Neurotrophin 4 | NT4P, Ntf5 |
| NM_012731 | Ntrk2 | Neurotrophic tyrosine kinase, receptor, type 2 | RATTRKB1, TRKB1, Tkrb, trk-B, trkB |
| NM_022868 | Pcdh8 | Protocadherin 8 | Arcadlin |
| NM_053460 | Pick1 | Protein interacting with PRKCA 1 | Prkcabp |
| NM_017034 | Pim1 | Pim-1 oncogene | - |
| NM_013151 | Plat | Plasminogen activator, tissue | PATISS, tPA |
| NM_013187 | Plcg1 | Phospholipase C, gamma 1 | PPLCA |
| NM_031527 | Ppp1ca | Protein phosphatase 1, catalytic subunit, alpha isoform | PP1alpha |
| NM_022498 | Ppp1cc | Protein phosphatase 1, catalytic subunit, gamma isoform | PP-1G, Ppp1cc1 |
| NM_130403 | Ppp1r14a | Protein phosphatase 1, regulatory (inhibitor) subunit 14A | Cpi17 |
| NM_017039 | Ppp2ca | Protein phosphatase 2, catalytic subunit, alpha isoform | Pp2a1 |
| NM_017041 | Ppp3ca | Protein phosphatase 3, catalytic subunit, alpha isoform | Calna1 |
| NM_001105713 | Prkca | Protein kinase C, alpha | Pkca |
| NM_012628 | Prkcg | Protein kinase C, gamma | PKC, PKCI, Prkc, Prkcc, RATPKCI |
| NM_001105731 | Prkg1 | Protein kinase, cGMP-dependent, type 1 | Pkgi, cGk1 |
| NM_013018 | Rab3a | RAB3A, member RAS oncogene family | RAB3 |
| NM_199267 | Rela | V-rel reticuloendotheliosis viral oncogene homolog A (avian) | NFkB |
| NM_080394 | Reln | Reelin | Reelen, Rl, reeler |
| XM_003750065 | Kif17 | Kinesin family member 17 | RGD1562511 |
| NM_053453 | Rgs2 | Regulator of G-protein signaling 2 | - |
| NM_013216 | Rheb | Ras homolog enriched in brain | - |
| NM_001107627 | Sirt1 | Sirtuin (silent mating type information regulation 2 homolog) 1 (S. cerevisiae) | Sir2 |
| NM_001109302 | Srf | Serum response factor (c-fos serum response element-binding transcription factor) | RGD1559787 |
| NM_021695 | Synpo | Synaptopodin | - |
| NM_053819 | Timp1 | TIMP metallopeptidase inhibitor 1 | TIMP-1, Timp |
| NM_012675 | Tnf | Tumor necrosis factor (TNF superfamily, member 2) | RATTNF, TNF-alpha, Tnfa |
| NM_013053 | Ywhaq | Tyrosine 3-monooxygenase/tryptophan 5-monooxygenase activation protein, theta polypeptide | 14-3-3t |
| NM_031144 | Actb | Actin, beta | Actx |
| NM_012512 | B2m | Beta-2 microglobulin | - |
| NM_012583 | Hprt1 | Hypoxanthine phosphoribosyltransferase 1 | Hgprtase, Hprt |
| NM_017025 | Ldha | Lactate dehydrogenase A | Ldh1 |
| NM_001007604 | Rplp1 | Ribosomal protein, large, P1 | - |
