# Supplementary material for: microRNA Expression Profiles in the Ventral Hippocampus during Pubertal Development and the Impact of Peri-Pubertal Binge Alcohol Exposure
Source: Noncoding RNA. 2019 Mar 5;5(1):21. doi: 10.3390/ncrna5010021 (PMC6468757; doi:10.3390/ncrna5010021)
Supplement: Supplementary file 1 [file ncrna-05-00021-s001.zip › ncrna-434944-suppl/S3 qPCR primers.docx]

| **Gene** | **Primer Sequence (5’ – 3’)** |
| --- | --- |
| rArc-F | TCAGACCATCACAGAACACCT |
| rArc-R | CCTTGGGTTTGGTGCCTACTT |
| rAR-F | AAAAGAGCTGCGGAAGGGAA |
| rAR-R | TTTCCGGAGACGACACGATG |
| rAtxn1-F | GAGCCAGCCAGACAGTGAAA |
| rAtxn1-R | TCATTGCTCCGCTCTTGGTT |
| rBace1-F | TCACCAATCAGTCCTTCCGC |
| rBace1-R | TAACGGTGCCTGTGGATGAC |
| rBC1-F | CGGTCCTCAGCTCCGAAAAA |
| rBC1 -R | GGTTGTGTGTGCCAGTTACC |
| rBDNF-F | AGCCTCCTCTGCTCTTTCTGCTGGA |
| rBDNF- R | GTTTGTCTATGCCCCTGCAGCCTT |
| rCRHR1-F | CACCTGGGCGCAGATCA |
| rCRHR1-R | CCTGGATCGCTCCGACATC |
| rDpysl2-F | GCCCTAGCTGGATCTGTGTT |
| rDpysl2-R | ATCCCTTAGCTGGTCTTGCT |
| rGrin2c-F | CAACGTCTTGGTTCCCCTCA |
| rGrin2c-R | GTTGAAGCCCCAAGAGACCA |
| rIL1B - F | CGACAAAATCCCTGTGGCCT |
| rIL1B - R | TGTTTGGGATCCACACTCTTC |
| rJunb-F | AGGCAGCTACTTTTCGGGTC |
| rJunb-R | TTGCTGTTGGGGACGATCAA |
| rKcnc3-F | TTGAAACCAACAGGGCAGAC |
| rKcnc3-R | ATCGGGCTCTTGTCTTCTGG |
| rMeCP2-F | GGGCTCAGGGAGGAAAAGTC |
| rMeCP2 - R | CACGAATGATGGAACGTCGC |
| rMmp9-F | GCTATGGTTACACTCGGGCA |
| rMmp9-R | TGGCCTTTAGTGTCTCGCTG |
| rNav3-F | AGAGAAGCGTTCGACCACAG |
| rNav3-R | ATCGACGTGGCTGTCCAAAT |
| rno-miR-19a-3p | CGC GTG TGC AAA TCT ATG CAA AAC TG |
| rno-miR-19b-3p | GCG TGT GCA AAT CCA TGC AAA ACT GA |
| rno-miR-29a-3p | GC GTA GCA CCA TCT GAA ATC GGT TA |
| rno-miR-29c-3p | CGC GTA GCA CCA TTT GAA ATC GGT TA |
| rno-miR-34a | CGTGG CAG TGT CTT AGC TGG TTG T |
| rno-miR-488-3p | GCG TTG AAA GGC TGT TTC TTG GTC |
| rNotch1-F | TTGGTCCGAGGGCATCTCTA |
| rNotch1-R | ACAGAGCTTGGGAACGGAAG |
| rNtf4-F | AGGCACTGGCTCTCAGAATG |
| rNtf4-R | CAAGCGGTGTCGATCCGAA |
| rPick1-F | TTCTCTGTGATTGGGGTGCG |
| rPick1-R | CCGAACTTCTCAATGCTGCG |
| rPim1-F | ACAACTCATTCCAGGCTCCG |
| rPim1-R | TGAGTCTGTGAGGGGCAAAG |
| rPOMC-F | CGACGGAGGAGAAAAGAGGTT |
| rPOMC-R | CTGAGGCTCTGTCGCGGAA |
| rRbm3-F | TAGAGGTGGTGGAGACCAGG |
| rRbm3 - R | TCTCTAGACCGCCCATACCC |
| rRplp1-F | GCATCTACTCCGCCCTCATC |
| rRplp1-R | GAGCCTTTGCAAACAAGCCA |
| rShank3-F | CTCTGAAGCCATTGGTCGGT |
| rShank3-R | AGGATCCAAGGGTTTGCCAG |
| rTNF-F | ATGGGCTCCCTCTCATCAGT |
| rTNF-R | GCTTGGTGGTTTGCTACGAC |
| rU87-F | CAATGATGACTTATGTTTTTGCCGT |
| rU87-R | GCTCAGTCTTAAGATTCTCTCTTCA |
| rVamp2-F | ATCTTTCAGCCCCCTCCCTT |
| rVamp2-R | AGCTGGCTATTTACAGGGGG |
| rVdac1-F | GTCACCGCCTCCGAGAACAT |
| rVdac1-R | CCGTAGCCCTTGGTGAAGAC |
| rYbx1 –F | CCCTGTGCAAGGAGAAGTGA |
| rYbx1 –R | CTGCGGAATCGTGGTCTGTA |
| rYbx3 –F | GCAAGTATCTGCGCAGTGTG |
| rYbx3 - R | ATCAGCAGCATAGCGACTCC |
