# Supplementary material for: microRNA Expression Profiles in the Ventral Hippocampus during Pubertal Development and the Impact of Peri-Pubertal Binge Alcohol Exposure
Source: Noncoding RNA. 2019 Mar 5;5(1):21. doi: 10.3390/ncrna5010021 (PMC6468757; doi:10.3390/ncrna5010021)
Supplement: Supplementary file 1 [file ncrna-05-00021-s001.zip › ncrna-434944-suppl/S4 Antibodies.docx]

| **Protein Target** | **Company** | **Catalog #** | **Dilution Used** |
| --- | --- | --- | --- |
| VDAC1 | Proteintech | 10866-1-AP | 1:1000 |
| KCNC3 | Abcam | Ab128832 | 1:1000 |
| ATXN1 | Novus Biologicals | NBP1-28734 | 1:1000 |
| VAMP2 | Novus Biologicals | AF5136 | 1:200 |
| β-TUBULIN | Cell Signaling | 2128S | 1:2000 |
