# Supplementary material for: microRNA Expression Profiles in the Ventral Hippocampus during Pubertal Development and the Impact of Peri-Pubertal Binge Alcohol Exposure
Source: Noncoding RNA. 2019 Mar 5;5(1):21. doi: 10.3390/ncrna5010021 (PMC6468757; doi:10.3390/ncrna5010021)
Supplement: Supplementary file 1 [file ncrna-05-00021-s001.zip › ncrna-434944-suppl/Table S1 miRNA array gene list.pdf]

miRBase or NCBI Accession No.	Mature miRNA ID or Gene Symbol
MIMAT0000775	rno-let-7b-5p
MIMAT0000776	rno-let-7c-5p
MIMAT0000562	rno-let-7d-5p
MIMAT0000777	rno-let-7e-5p
MIMAT0000779	rno-let-7i-5p
MIMAT0000615	rno-miR-101b-3p
MIMAT0012825	rno-miR-105
MIMAT0000825	rno-miR-106b-5p
MIMAT0000826	rno-miR-107-3p
MIMAT0000828	rno-miR-124-3p
MIMAT0000830	rno-miR-125b-5p
MIMAT0000831	rno-miR-126a-5p
MIMAT0000834	rno-miR-128-3p
MIMAT0000836	rno-miR-130a-3p
MIMAT0000838	rno-miR-132-3p
MIMAT0003126	rno-miR-133b-3p
MIMAT0000840	rno-miR-134-5p
MIMAT0000844	rno-miR-138-5p
MIMAT0000845	rno-miR-139-5p
MIMAT0000573	rno-miR-140-5p
MIMAT0000849	rno-miR-143-3p
MIMAT0000852	rno-miR-146a-5p
MIMAT0005595	rno-miR-146b-5p
MIMAT0000579	rno-miR-148b-3p
MIMAT0000853	rno-miR-150-5p
MIMAT0000614	rno-miR-151-3p
MIMAT0000854	rno-miR-152-3p
MIMAT0000784	rno-miR-15b-5p
MIMAT0000858	rno-miR-181a-5p
MIMAT0000857	rno-miR-181c-5p
MIMAT0005299	rno-miR-181d-5p
MIMAT0000866	rno-miR-191a-5p
MIMAT0000869	rno-miR-194-5p
MIMAT0000870	rno-miR-195-5p
MIMAT0000789	rno-miR-19a-3p
MIMAT0000788	rno-miR-19b-3p
MIMAT0000876	rno-miR-203a-3p
MIMAT0000602	rno-miR-20a-5p
MIMAT0003211	rno-miR-20b-5p
MIMAT0000883	rno-miR-212-3p
MIMAT0000791	rno-miR-22-3p
MIMAT0000792	rno-miR-23a-3p
MIMAT0000794	rno-miR-24-3p
MIMAT0000797	rno-miR-26b-5p
MIMAT0000799	rno-miR-27a-3p
MIMAT0000800	rno-miR-28-5p
MIMAT0000900	rno-miR-298-5p
MIMAT0000802	rno-miR-29a-3p
MIMAT0000801	rno-miR-29b-3p
MIMAT0000803	rno-miR-29c-3p
MIMAT0000808	rno-miR-30a-5p
MIMAT0000807	rno-miR-30d-5p
MIMAT0000805	rno-miR-30e-5p
MIMAT0000812	rno-miR-33-5p
MIMAT0000577	rno-miR-337-3p
MIMAT0000581	rno-miR-338-3p
MIMAT0000583	rno-miR-339-5p
MIMAT0000589	rno-miR-342-3p

MIMAT0000596	rno-miR-346
MIMAT0000815	rno-miR-34a-5p
MIMAT0003117	rno-miR-361-5p
MIMAT0003196	rno-miR-376b-3p
MIMAT0003199	rno-miR-381-3p
MIMAT0003201	rno-miR-382-5p
MIMAT0003205	rno-miR-409a-3p
MIMAT0001626	rno-miR-431
MIMAT0001628	rno-miR-433-3p
MIMAT0005316	rno-miR-455-5p
MIMAT0005319	rno-miR-484
MIMAT0003203	rno-miR-485-5p
MIMAT0005341	rno-miR-488-3p
MIMAT0003113	rno-miR-489-3p
MIMAT0003381	rno-miR-499-5p
MIMAT0012829	rno-miR-511-5p
MIMAT0003176	rno-miR-539-5p
MIMAT0005325	rno-miR-598-3p
MIMAT0005342	rno-miR-652-3p
MIMAT0000606	rno-miR-7a-5p
MIMAT0000781	rno-miR-9a-5p
MIMAT0004708	rno-miR-9a-3p
MIMAT0000816	rno-miR-92a-3p
MIMAT0005340	rno-miR-92b-3p
MIMAT0000817	rno-miR-93-5p
MIMAT0000819	rno-miR-98-5p
MIMAT0000010	cel-miR-39-3p
MIMAT0000010	cel-miR-39-3p
	SNORD61
	SNORD68
	SNORD72
	SNORD95
	SNORD96A
	RNU6-6P
