# Supplementary material for: microRNA Expression Profiles in the Ventral Hippocampus during Pubertal Development and the Impact of Peri-Pubertal Binge Alcohol Exposure
Source: Noncoding RNA. 2019 Mar 5;5(1):21. doi: 10.3390/ncrna5010021 (PMC6468757; doi:10.3390/ncrna5010021)
Supplement: Supplementary file 1 [file ncrna-05-00021-s001.zip › ncrna-434944-suppl/Table S2 mRNA array gene list.pdf]

GeneBank	Symbol	Description	Gene Name
NM_019254	Adam10	ADAM metallopeptidase domain 10	MADM
NM_001107239	Adcy1	Adenylate cyclase 1 (brain)	Ac1
NM_017142	Adcy8	Adenylate cyclase 8 (brain)	Ac8
NM_033230	Akt1	V-akt murine thymoma viral oncogene homolog 1	Akt
NM_019361	Arc	Activity-regulated cytoskeleton-associated protein	rg3.1
NM_012513	Bdnf	Brain-derived neurotrophic factor	-
NM_012920	Camk2a	Calcium/calmodulin-dependent protein kinase II alpha	PK2CDD, PKCCD
NM_133605	Camk2g	Calcium/calmodulin-dependent protein kinase II gamma	-
NM_031333	Cdh2	Cadherin 2	N-cadherin
NM_024125	Cebpb	CCAAT/enhancer binding protein (C/EBP), beta	Il6dbp, NF-IL6, TCF5
NM_013154	Cebpd	CCAAT/enhancer binding protein (C/EBP), delta	C, EBPd, CELF
NM_012784	Cnr1	Cannabinoid receptor 1 (brain)	SKR6R
NM_031017	Creb1	CAMP responsive element binding protein 1	Creb
NM_001110860	Crem	CAMP responsive element modulator	Icer
NM_019621	Dlg4	Discs, large homolog 4 (Drosophila)	Dlgh4, PSD95, Sap90
NM_012551	Egr1	Early growth response 1	Krox-24, NGFI-A, Ngf1, Ngfi, zif-268
NM_053633	Egr2	Early growth response 2	Krox20
NM_017086	Egr3	Early growth response 3	-
NM_019137	Egr4	Early growth response 4	Egr4l1, NGFI-C
NM_001127319	Ephb2	Eph receptor B2	RGD1564232
NM_022197	Fos	FBJ osteosarcoma oncogene	c-fos
NM_017295	Gabra5	Gamma-aminobutyric acid (GABA) A receptor, alpha 5	-
NM_013145	Gnai1	Guanine nucleotide binding protein (G protein), alpha inhibiting 1	BPGTPB
NM_031608	Gria1	Glutamate receptor, ionotropic, AMPA 1	GluA1, gluR-A
NM_017261	Gria2	Glutamate receptor, ionotropic, AMPA 2	GluA2, GluR-K2, GluR2, gluR-B
NM_032990	Gria3	Glutamate receptor, ionotrophic, AMPA 3	GLUR3, GluA3, GluR-3, GluR-C, GluR-K3
NM_017263	Gria4	Glutamate receptor, ionotrophic, AMPA 4	GluA4, GluR-D, GluR4
NM_017010	Grin1	Glutamate receptor, ionotropic, N-methyl D-aspartate 1	GluN1, NMDAR1, NR1
NM_012573	Grin2a	Glutamat receptor, ionotropic, N-methyl D-aspartate 2A	GluN2A, NMDAR2A, NR2A
NM_012574	Grin2b	Glutamate receptor, ionotropic, N-methyl D-aspartate 2B	GluN2B
NM_012575	Grin2c	Glutamate receptor, ionotropic, N-methyl D-aspartate 2C	GluN2C, NR2C
NM_022797	Grin2d	Glutamate receptor, ionotropic, N-methyl D-aspartate 2D	GluN2D
NM_032069	Grip1	Glutamate receptor interacting protein 1	-
NM_017011	Grm1	Glutamate receptor, metabotropic 1	Gprc1a
NM_001105711	Grm2	Glutamate receptor, metabotropic 2	-
NM_001105712	Grm3	Glutamate receptor, metabotropic 3	mGluR3
NM_022666	Grm4	Glutamate receptor, metabotropic 4	-
NM_017012	Grm5	Glutamate receptor, metabotropic 5	mGluR5, mGlur5
NM_031040	Grm7	Glutamate receptor, metabotropic 7	-
NM_022202	Grm8	Glutamate receptor, metabotropic 8	Glur8, Gprc1h, Mglur8, mGluR8b, mGlur
NM_031707	Homer1	Homer homolog 1 (Drosophila)	HOMER1F, Vesl-1
NM_178866	Igf1	Insulin-like growth factor 1	-
NM_017128	Inhba	Inhibin beta-A	-
NM_021835	Jun	Jun oncogene	-
NM_021836	Junb	Jun B proto-oncogene	-
NM_031135	Klf10	Kruppel-like factor 10	Tieg
NM_053842	Mapk1	Mitogen activated protein kinase 1	ERK-2, ERT1, Erk2, p42-MAPK
NM_031055	Mmp9	Matrix metallopeptidase 9	-
NM_031521	Ncam1	Neural cell adhesion molecule 1	Cd56, N-CAM, N-CAM-1, NCAM-1, NCAM-C, NCAMC, Ncam
NM_001276711	Nfkb1	Nuclear factor of kappa light polypeptide gene enhancer in B-cells 1	EBP-1, NF-kB
NM_030867	Nfkbib	Nuclear factor of kappa light polypeptide gene enhancer in B-cells inhibitor, beta	-

NM_001277055	Ngf	Nerve growth factor (beta polypeptide)	Ngfb, beta-NGF
NM_012610	Ngfr	Nerve growth factor receptor (TNFR superfamily, member 16)	LNGFR, RNNGFRR, Tnfrsf16, p75, p75NTR
NM_052799	Nos1	Nitric oxide synthase 1, neuronal	bNOS
NM_001034199	Nptx2	Neuronal pentraxin 2	NP-II, NP2, Narp
NM_024388	Nr4a1	Nuclear receptor subfamily 4, group A, member 1	HMR, Ngfi-b, Nur77
NM_031073	Ntf3	Neurotrophin 3	-
NM_013184	Ntf4	Neurotrophin 4	NT4P, Ntf5
NM_012731	Ntrk2	Neurotrophic tyrosine kinase, receptor, type 2	RATTRKB1, TRKB1, Tkrb, trk-B, trkB
NM_022868	Pcdh8	Protocadherin 8	Arcadlin
NM_053460	Pick1	Protein interacting with PRKCA 1	Prkcabp
NM_017034	Pim1	Pim-1 oncogene	-
NM_013151	Plat	Plasminogen activator, tissue	PATISS, tPA
NM_013187	Plcg1	Phospholipase C, gamma 1	PPLCA
NM_031527	Ppp1ca	Protein phosphatase 1, catalytic subunit, alpha isoform	PP1alpha
NM_022498	Ppp1cc	Protein phosphatase 1, catalytic subunit, gamma isoform	PP-1G, Ppp1cc1
NM_130403	Ppp1r14a	Protein phosphatase 1, regulatory (inhibitor) subunit 14A	Cpi17
NM_017039	Ppp2ca	Protein phosphatase 2, catalytic subunit, alpha isoform	Pp2a1
NM_017041	Ppp3ca	Protein phosphatase 3, catalytic subunit, alpha isoform	Calna1
NM_001105713	Prkca	Protein kinase C, alpha	Pkca
NM_012628	Prkcg	Protein kinase C, gamma	PKC, PKCI, Prkc, Prkcc, RATPKCI
NM_001105731	Prkg1	Protein kinase, cGMP-dependent, type 1	Pkgi, cGk1
NM_013018	Rab3a	RAB3A, member RAS oncogene family	RAB3
NM_199267	Rela	V-rel reticuloendotheliosis viral oncogene homolog A (avian)	NFkB
NM_080394	Reln	Reelin	Reelen, Rl, reeler
XM_003750065	Kif17	Kinesin family member 17	RGD1562511
NM_053453	Rgs2	Regulator of G-protein signaling 2	-
NM_013216	Rheb	Ras homolog enriched in brain	-
NM_001107627	Sirt1	Sirtuin (silent mating type information regulation 2 homolog) 1 (S. cerevisiae)	Sir2
NM_001109302	Srf	Serum response factor (c-fos serum response element-binding transcription factor)	RGD1559787
NM_021695	Synpo	Synaptopodin	-
NM_053819	Timp1	TIMP metallopeptidase inhibitor 1	TIMP-1, Timp
NM_012675	Tnf	Tumor necrosis factor (TNF superfamily, member 2)	RATTNF, TNF-alpha, Tnfa
NM_013053	Ywhaq	Tyrosine 3-monooxygenase/tryptophan 5-monooxygenase activation protein, theta polypeptide	14-3-3t
NM_031144	Actb	Actin, beta	Actx
NM_012512	B2m	Beta-2 microglobulin	-
NM_012583	Hprt1	Hypoxanthine phosphoribosyltransferase 1	Hgprtase, Hprt
NM_017025	Ldha	Lactate dehydrogenase A	Ldh1
NM_001007604	Rplp1	Ribosomal protein, large, P1	-
