# Supplementary material for: microRNA Expression Profiles in the Ventral Hippocampus during Pubertal Development and the Impact of Peri-Pubertal Binge Alcohol Exposure
Source: Noncoding RNA. 2019 Mar 5;5(1):21. doi: 10.3390/ncrna5010021 (PMC6468757; doi:10.3390/ncrna5010021)
Supplement: Supplementary file 1 [file ncrna-05-00021-s001.zip › ncrna-434944-suppl/Table S3 qPCR primers.pdf]

Gene	Primer Sequence (5' – 3')
rArc-F	TCAGACCATCACAGAACACCT
rArc-R	CCTTGGGTTTGGTGCCTACTT
rAR-F	AAAAGAGCTGCGGAAGGGAA
rAR-R	TTTCCGGAGACGACACGATG
rAtxn1-F	GAGCCAGCCAGACAGTGAAA
rAtxn1-R	TCATTGCTCCGCTCTTGGTT
rBace1-F	TCACCAATCAGTCCTTCCGC
rBace1-R	TAACGGTGCCTGTGGATGAC
rBC1-F	CGGTCCTCAGCTCCGAAAAA
rBC1 -R	GGTTGTGTGTGCCAGTTACC
rBDNF-F	AGCCTCCTCTGCTCTTTCTGCTGGA
rBDNF- R	GTTTGTCTATGCCCCTGCAGCCTT
rCRHR1-F	CACCTGGGCGCAGATCA
rCRHR1-R	CCTGGATCGCTCCGACATC
rDpysl2-F	GCCCTAGCTGGATCTGTGTT
rDpysl2-R	ATCCCTTAGCTGGTCTTGCT
rGrin2c-F	CAACGTCTTGGTTCCCCTCA
rGrin2c-R	GTTGAAGCCCCAAGAGACCA
rIL1B - F	CGACAAAATCCCTGTGGCCT
rIL1B - R	TGTTTGGGATCCACACTCTTC
rJunb-F	AGGCAGCTACTTTTCGGGTC
rJunb-R	TTGCTGTTGGGGACGATCAA
rKcnc3-F	TTGAAACCAACAGGGCAGAC
rKcnc3-R	ATCGGGCTCTTGTCTTCTGG
rMeCP2-F	GGGCTCAGGGAGGAAAAGTC
rMeCP2 - R	CACGAATGATGGAACGTCGC
rMmp9-F	GCTATGGTTACACTCGGGCA
rMmp9-R	TGGCCTTTAGTGTCTCGCTG
rNav3-F	AGAGAAGCGTTCGACCACAG
rNav3-R	ATCGACGTGGCTGTCCAAAT
rno-miR-19a-3p	CGC GTG TGC AAA TCT ATG CAA AAC TG
rno-miR-19b-3p	GCG TGT GCA AAT CCA TGC AAA ACT GA
rno-miR-29a-3p	GC GTA GCA CCA TCT GAA ATC GGT TA
rno-miR-29c-3p	CGC GTA GCA CCA TTT GAA ATC GGT TA
rno-miR-34a	CGTGG CAG TGT CTT AGC TGG TTG T
rno-miR-488-3p	GCG TTG AAA GGC TGT TTC TTG GTC
rNotch1-F	TTGGTCCGAGGGCATCTCTA
rNotch1-R	ACAGAGCTTGGGAACGGAAG
rNtf4-F	AGGCACTGGCTCTCAGAATG
rNtf4-R	CAAGCGGTGTCGATCCGAA
rPick1-F	TTCTCTGTGATTGGGGTGCG
rPick1-R	CCGAACTTCTCAATGCTGCG
rPim1-F	ACAACTCATTCCAGGCTCCG
rPim1-R	TGAGTCTGTGAGGGGCAAAG
rPOMC-F	CGACGGAGGAGAAAAGAGGTT
rPOMC-R	CTGAGGCTCTGTCGCGGAA
rRbm3-F	TAGAGGTGGTGGAGACCAGG
rRbm3 - R	TCTCTAGACCGCCCATACCC
rRplp1-F	GCATCTACTCCGCCCTCATC
rRplp1-R	GAGCCTTTGCAAACAAGCCA
rShank3-F	CTCTGAAGCCATTGGTCGGT
rShank3-R	AGGATCCAAGGGTTTGCCAG
rTNF-F	ATGGGCTCCCTCTCATCAGT
rTNF-R	GCTTGGTGGTTTGCTACGAC
rU87-F	CAATGATGACTTATGTTTTTGCCGT
rU87-R	GCTCAGTCTTAAGATTCTCTCTTCA
rVamp2-F	ATCTTTCAGCCCCCTCCCTT
rVamp2-R	AGCTGGCTATTTACAGGGGG

rVdac1-F	GTCACCGCCTCCGAGAACAT
rVdac1-R	CCGTAGCCCTTGGTGAAGAC
rYbx1 –F	CCCTGTGCAAGGAGAAGTGA
rYbx1 –R	CTGCGGAATCGTGGTCTGTA
rYbx3 –F	GCAAGTATCTGCGCAGTGTG
rYbx3 - R	ATCAGCAGCATAGCGACTCC
