# Supplementary material for: microRNA Expression Profiles in the Ventral Hippocampus during Pubertal Development and the Impact of Peri-Pubertal Binge Alcohol Exposure
Source: Noncoding RNA. 2019 Mar 5;5(1):21. doi: 10.3390/ncrna5010021 (PMC6468757; doi:10.3390/ncrna5010021)
Supplement: Supplementary file 1 [file ncrna-05-00021-s001.zip › ncrna-434944-suppl/Table S4 Antibodies.pdf]

Protein Target	Company	Catalog #	Dilution Used
VDAC1	Proteintech	10866-1-AP	1:1000
KCNC3	Abcam	Ab128832	1:1000
ATXN1	Novus Biologicals	NBP1-28734	1:1000
VAMP2	Novus Biologicals	AF5136	1:200
β-TUBULIN	Cell Signaling	2128S	1:2000
